# Supplementary material for: Histopathology and immunohistochemistry as prognostic factors for poorly differentiated thyroid cancer in a series of Polish patients
Source: PLoS One. 2020 Feb 24;15(2):e0229264. doi: 10.1371/journal.pone.0229264 (PMC7039429; doi:10.1371/journal.pone.0229264)
Supplement: Attachment — Submitted filename: Response_to_Reviewers.docx [file pone.0229264.s001.docx]

Kielce, 21/JAN/2020

Professor Jason Chia-Hsun Hsieh,

Academic Editor

PLOS ONE

Dear Sir,

On behalf of myself and all co-authors, I would like to thank you for considering our manuscript ‘Histopathology and Immunohistochemistry as Prognostic Factors for Poorly Differentiated Thyroid Cancer in a Series of Polish Patients’ for publication in PLOS ONE. According to the journal requirements, we are sending the revised version of our paper in two files: ‘Revised Manuscript with Track Changes’ and ‘Manuscript’. The rebuttal letter with responses to each points raised by the Academic Editor and the Reviewers’ comments is below.

**Academic Editor:**

On behalf of myself and all co-authors, I would like to thank you for your revision and the constructive guidelines and comments, which will let us improve the manuscript to be accepted for the publication in PLOS ONE.

Question 1.

The revised manuscript was improved according PLOS ONE’s style templates to meet the journal requirements which were presented in particular guidelines for Authors.

Question 2.

All the tables were re-designed according to the PLOS ONE’s formatting rules and included to the revised manuscript after paragraphs in which they had been cited first. Additionally, all the figures captions were included in the relevant places of the revised manuscript, but the figure files were submitted in individual files in formats ‘.tiff’ according to the PLOS ONE’s formatting rules.

Question 3.

The ethics statement was extended to the statement that all the initial data as well as follow-up data were analysed anonymously as well as the ethics committee waived the requirement for informed consent (line 92-95). We also highlighted that pathologists were blinded to the clinical data and outcome (line 123). In regard to the sentence about results of IHC analyses we added again the clear statement that the follow-up data were analysed anonymously including a medical record of a patient without IHC analysis but with known clinical outcome (line 232-234).

Question 4.

Unfortunately, your HTML markup was not attached to your post. However, we scanned our manuscript by an overlapping program and finally we found few sentences in the Methods section and improved them. According to the cited by pathologists protocol of mitotic rate and Ki-67 in the Methods section [identified by you as <https://doi.org/10.1038/sj.bjc.6601453>] in line 136 and 162, we added a new reference in that paragraphs as 18^th^ position. So, all the references cited after [REF18] were re-counted and ordered *de novo.*

We also added REF 6 [identified by you as <https://doi.org/10.1038/modpathol.2010.117>] in line 153. We cited our own work [REF 15; identified by you as <https://doi.org/10.1111/cen.13910>], in the Methods section, line 99. Outside this section we rephrased the text of our previous work in lines 182-183, 191-196 in the Results section. We also found that the sentence in lines 139-140 in the Methods section requires revision and we rephrased it.

The overlapping program did not indicate any more overlapping text outside the Methods section which should have been revised.

The additional authors’ remark: in the revised manuscript an update in the funding statement was included, according to the updated Jan Kochanowski University in Kielce requirements. We also changed the affiliations of two co-authors (SG, AK) into the updated name: ‘Collegium Medicum’, Jan Kochanowski University, Kielce.

**Reviewer 1:**

On behalf of myself and all co-authors, I would like to thank you for your revision of our manuscript and the constructive guidelines and comments, which will let us improve the manuscript to be accepted for the publication in PLOS ONE.

Question 1.

The incidence of PDTC is not reported as being similar to other different studies. The recent WHO classification of endocrine tumors [REF 12 in manuscript] indicates that the frequency of PDTC varies from less than 1% (e.g. in Japan) to 6.7% in Northern Italy. However, it is worth noting that the lower percentage of PDTC is usually reported in populations of sufficient iodine supply. Firstly, in 2007, Sanders et al. in their well-known study put our attention on the potential impact of environmental, e.g. related to dietary factors (including iodine), or some genetic factors on the occurrence of PDTC in the patients’ population from different areas. In Northern Italy, which was a region of a long-term insufficient supply of iodine, the occurrence was reported to be up to 15%, however, a later report showed a smaller proportion of PDTC, accounting for only 6.7% in a number of Italian patients [Asioli et al., 2010; REF 6 in manuscript]. Eventually, the WHO indicates a rate of 6.7% as being the highest reported figure from Europe.

The Reviewer cites two studies based on PDTC patients from the USA, which is a leading country of sufficient iodine supplementation on a population level, and comments that the prevalence ratio was 6% from the US data. We would kindly like to remark, that in the first cited study published by Landa et al., we did not find any piece of information about the frequency of PDTC in the American population, despite the fact there was a very interesting analysis of the mutational status of 117 thyroid cancers containing 84 PDTC cases. In the second, by Fagin et al., we can indeed find the Authors’ note about the PDTC prevalence rate of 6%, but it is worth noting that the paper was a review, not an original study, and the Authors do not indicate any reference to the source of this fact. In summary, we do not find any evidence for such a high prevalence of 6% PDTC-patients from the USA, which is a country of long-term sufficient iodine supply.

So far, the occurrence of PDTC in the USA, was reported as low as 1.8% (56/3128) in the original study by Asioli et al. from the well-known Mayo Clinic, in the USA, in which the Authors identified PDTC on the basis of the Turin criteria according to the recommendations by WHO in 2004 and 2018. The rate of 1.8% is indicated by the recent WHO classification as an evidenced rate of PDTC in patients from the USA. Additionally, we would like to note that Poland has been listed as an European country of sufficient iodine supply for close to 20 years after the introduction of the mandatory iodine prophylaxis program in 1997. This fact had been documented by the WHO in nationwide population-based studies performed in 2003 under the supervision of the International Council for the Control of Iodine Deficiency Disorders (ICCIDD; now Iodine Global Network [IGN]; http://ign.org/) and its Polish branch, and published by WHO in 2007 [Andersson M, de Benoist B et al. (eds) 2007 Iodine Deficiency in Europe, WHO]. In view of this data, our result of 1.89% of PDTC in Polish patients remains in accordance with the US data.

Despite the consideration above, we can agree with the Reviewer’s main remark, that the variation of PDTC incidence is an established fact, and the potentially causing factors have been added (line 57-59) to the revised manuscript.

It is agreed that the pathologists can identify PDTC, however its histological definition is still under debate among pathologists. The use of the MSKCC criteria may probably better identify PDTC patients of high risk rather than the use of the Turin criteria. However, the recent WHO classification does not recommend a diagnosis of PDTC in thyroid tumors that meet the MSKCC criteria, but do not meet the Turin criteria. This statement is clearly written in the manuscript (line 74-76). The prevalence of PDTC in Poland is reported as based on the Turin criteria, according to the WHO recommendations. The MSKCC criteria was proposed on the basis of proliferate grading and that’s why they are known as more restrictive than the Turin criteria. The use of the MSKCC criteria will most likely decrease the amount of PDTC. The incidence of PDTC will be lower than the reported 1.89% (based on the Turin criteria) which are an expected result of the use of more restrictive pathological criteria with subsequent potential consequences being an unreliable analyses of very low subgroups. Even though, such analysis has not been performed, we would like to bring the Reviewer’s attention to the fact that the present study was designed on the basis of the present WHO definition of PDTC which still recommends the use of the Turin criteria.

Question 2.

Unfortunately, the mutational status of studied PDTC cases was not performed due to a financial aspect. We agree with the Reviewer’s remark, that such analysis would be very interesting, but it was not the aim of the current study. However, we consider it and we perform it willingly provided that the authors’ institution fund it.

According to the Reviewer’s comment we agree that the potential clinical usefulness of a presence of atypical mitoses or Tg immunostaining in surgery report of PDTC should be more widely discussed. In our opinion this statement is more highlighted in the revised manuscript (lines 354-363), however a few comments were written in the primary version of the manuscript.

**Reviewer 2.**

On behalf of myself and all co-authors, I would like to thank you for your revision of our manuscript and the constructive guidelines and comments, which will let us improve the manuscript to be accepted for the publication in PLOS ONE.

Question 1.

We agree that the sample size is small. However, we mention that all subsequent patients with PDTC who came to our center between 2000 and 2018 were included, so it is not possible to increase the sample size to obtain the power of the tests at 80% level. Below we present the post-hoc calculations power of tests for the results included in Table 3. In that calculations, HR from Table 3 was taken as the assumed value of the effect. In addition, we also present the calculated sample sizes that would be necessary to achieve test power at 80%. All calculations were made using R statistical software with powerSurvEpi package.

Nevertheless, we mention that post-hoc calculations for test power (and especially the use of the observed effect value) are criticized by some authors (see e.g. [https://journals.lww.com/annalsofsurgery/Citation/2019/01000/Don_t_Calculate_Post_hoc_Power_Using_Observed .46.aspx](https://journals.lww.com/annalsofsurgery/Citation/2019/01000/Don_t_Calculate_Post_hoc_Power_Using_Observed%20.46.aspx) ).

| **Variable** | **Variable**  **levels** | **HR**  **(point estimate)** | **Power of test**  **(post-hoc calculations)** | **Sample size needed**  **for power of test = 80%** |
| --- | --- | --- | --- | --- |
| Age, yrs | < 55 | Ref. level | 0.03 | 19665 |
|  | >=55 | 1.04 |  | 37016 |
| Gender | Female | Ref. level | 0.06 | 1703 |
|  | Male | 1.2 |  | 826 |
| Tumor size, cm | <=4 | Ref. level | 0.63 | 29 |
|  | >4 | 3.9 |  | 44 |
| Pattern of PD growth | Solid | 1.4 | 0.09 | 237 |
|  | Insular | Ref. level |  | 672 |
| PD area | <=50% | Ref. level | 0.05 | 867 |
|  | >50% | 1.2 |  | 3292 |
| PD area | <=95% | Ref. level | 0.17 | 190 |
|  | >95% | 1.6 |  | 190 |
| Predominant insular pattern of growth | No | Ref. level | 0.04 | 3639 |
|  | Yes | 1.1 |  | 6065 |
| Presence of WD component | No | 1.6 | 0.17 | 190 |
|  | Yes | Ref. level |  | 190 |
| Presence of necrosis | No | Ref. level | 0.17 | 121 |
|  | Yes | 1.7 |  | 274 |
| Amount of necrosis | <=1% | Ref. level | 0.11 | 383 |
|  | >1% | 1.4 |  | 312 |
| Extensive necrosis > 5% | No | Ref. level | 0.13 | 412 |
|  | Yes | 1.5 |  | 149 |
| Convoluted nuclei | No | 0.6 | 0.23 | 92 |
|  | Yes | Ref. level |  | 173 |
| Mitosis >=3/10 high-power fields | No | Ref. level | 0.21 | 201 |
|  | Yes | 1.7 |  | 89 |
| Atypical mitoses | No | Ref. level | 0.72 | 38 |
|  | Yes | 3.2 |  | 22 |
| Extensive vascular invasion | No | Ref. level | 0.20 | 220 |
|  | Yes | 1.6 |  | 97 |
| Ki-67 > 5% | No | Ref. level | 0.15 | 47 |
|  | Yes | 2.8 |  | 404 |
| TTF-1 positive | No | Ref. level | 0.04 | 1607 |
|  | Yes | 0.9 |  | 11248 |
| p53 positive | No | Ref. level | 0.27 | 161 |
|  | Yes | 1.9 |  | 48 |
| Thyroglobulin positive | No | 3.3 | 0.73 | 14 |
|  | Yes | Ref. level |  | 44 |
| CK-19 positive | No | Ref. level | 0.04 | 6986 |
|  | Yes | 1.1 |  | 2595 |
| IMP3 positive | No | Ref. level | 0.07 | 1333 |
|  | Yes | 1.3 |  | 228 |

Based on <https://acsjournals.onlinelibrary.wiley.com/doi/full/10.1002/cncr.29924> small, medium, and large HRs comparing 2 groups would be approximately 1.3, 1.9, and 2.8.

In the case of the results given in Table 3, in many cases the effect measured with HR is small (i.e. below 1.3) and only in 4 cases HR is greater or equal than 2.8. In all of this 4 cases (tumor size> 4, atypical mitoses, Ki-67> 5%, thyroglobulin positive) HR was statistically significant in our group of patients, and therefore our sample size was sufficient to confirm the occurrence of that large effects.

Question 2.

The majority of the present group (46/49) was recruited to the study focused on PDTC with other objectives [REF 15 in manuscript] which was designed and performed in our institution previously, as we described in the Methods section [line 100]. In that study the main aim was to validate the recent 8^th^ Edition of AJCC/TNM staging system in a stratification of a risk of death of the patients with PDTC and evaluate the impact of the updated TNM stage on the patients’ survival. As we know, the major changes of the recent 8^th^ Ed AJCC/TNM staging system comprised of an increase in the cut-off age from 45 to 55 years of age at diagnosis, and the removal of lymph nodes metastases and minor extrathyroidal extension (ETE) from the definition of tumor primary pT3 stage and resulted in a general trend towards the downstaging of TNM staging system. Hence, according to the aim of the previous study, the prognostic role of ETE in different aspects: presence or absence of ETE in PDTC, ETE extent (minor/gross) and their impact on PDTC-patients’ survival has been already analyzed in our 46 cases of PDTC and reported in detail previously [REF 15 in manuscript]. We agree that the presence/absence of ETE is an obligatory element in a surgery report of thyroid cancer, but its prognostic role was validated in the basis of our PDTC and an extension of the current analysis to 49 cases will not likely change the result, and it could be an overlapping analysis, so we decided not to perform it again.

To summarise, we hope both the Reviewers and the Editorial Board will find the responses to the comments discussed above as satisfactory.

Yours sincerely,

Agnieszka Walczyk
